# Antitumor Organometallics. IV. The Mutagenic Potential of Some Diphenylantimony(III) Dithiophosphorus Derivatives

**DOI:** 10.1155/MBD.1994.291a

**Published:** 1994

**Authors:** Carmen Socaciu, Ioan Pasca, Cristian Silvestru, Adela Bara, Ionel Haiduc

**Affiliations:** 1 Department of Chemistry and Biochemistry University of Agricultural Sciences Cluj-Napoca Romania; 2 Chemistry Department "Babes-Bolyai" University Cluj-Napoca RO-3400 Romania; 3 Oncology Institute of Cluj-Napoca Cluj-Napoca Romania

## Abstract

Two diphenylantimony(III) derivatives of dithiophosphorus ligands, *i.e.* Ph_2_SbS_2_PPh_2_ and Ph_2_SbS_2_P(OPr-i)_2_, which were previously found to exhibit antitumor properties, have been now investigated for potential mutagenic effects in healthy and Ehrlich ascites tumor-bearing mice. Two short-term tests, *i.e.* the micronucleus test and the cytogenetic analysis, were used as end-points for mutagenicity. The results are consistent with a mutagenic potential for both organoantimony(III) compounds tested, the effect being higher for the phosphorodithioato derivative.


### ANTITUMOR ORGANOMETALLICS. IV. THE MUTAGENIC POTENTIAL OF SOME DIPHENYLANTIMONY(III) DITHIOPHOSPHORUS DERIVATIVES

Carmen Socaciu,[1] Ioan Pasca,[1] Cristian Silvestru,*[2]

Adela Bara,[3] and Ionel Haiduc[2]

[1] Department of Chemistry and Biochemistry, University of Agricultural Sciences, Cluj-Napoca
[2] Chemistry Department, "Babes-Bolyai" University, RO-3400 Cluj-Napoca
[3] Oncology Institute of Cluj-Napoca, Cluj-Napoca, Romania.

### ABSTRACT

Two diphenylantimony(III) derivatives of dithiophosphorus ligands, *i.e.* $Ph_2SbS_2PPh_2$ and $Ph_2SbS_2P(OPr\text{-}i)_2$, which were previously found to exhibit antitumor properties, have been now investigated for potential mutagenic effects in healthy and Ehrlich ascites tumor-bearing mice. Two short-term tests, *i.e.* the micronucleus test and the cytogenetic analysis, were used as end-points for mutagenicity. The results are consistent with a mutagenic potential for both organoantimony(III) compounds tested, the effect being higher for the phosphorodithioato derivative.

### INTRODUCTION

Cancer remains one of the major causes of death in humans and many efforts are in progress in the fight against this disease, including new treatments and new antitumor agents, *i.e.* metal compounds.[1-3] The use of chemotherapy in cancer treatment has always been hampered by the possible side effects of the drugs applied, irrespective of their antitumor effects.

The platinum compounds and especially Cisplatin are active antitumor drugs, but revealed systemic toxicity[4,5] and even mutagenicity.[6-8] The screening of anticancer agents includes the testing of their cytotoxic and genotoxic potential by *in vitro* and *in vivo* assays,[9-11] arranged in a logic succession, from *in vitro* short-term investigations to *in vivo* long-term experiments on successive animal generations[12,13] and clinical trials on humans.[9,12,14] Usually the first steps of the screening include the bacterial assays (Ames test,[9,14] SOS Chromotest[15]) and the cytogenetic analysis (the micronucleus test[13,16,17] and chromosome aberrations[13,18]).

During the last fiftheen years much attention was focussed on the antitumor properties of new organometallic compounds.[1,2] A large number of organotin derivatives, some of them being first investigated for their biocidal properties towards bacteria, fungi and mammals,[19] were found to exhibit antitumor activity.[1-3,20,21] Recently, two diphenylantimony(III) derivatives of dithiophosphorus ligands were mentioned as the first organoantimony compounds exhibiting antitumor properties.[22-25] The genotoxic properties of organoantimony compounds are practically not investigated, and, so far, only one inorganic antimony compound was reported to exhibit mutagenic potential against human lymphocytes.[26]

Our investigations, reported here, are focused on the mutagenic properties of the two diphenylantimony(III) derivatives previously tested fot their antitumor effects on animal tumor systems.[22-25] The micronucleus test and the cytogenetic analysis were used as sensible end-points for testing their mutagenicity. These assays were applied comparatively on organoantimony-treated, healthy or Ehrlich ascites tumor (EAT) bearing mice.

### MATERIALS AND METHODS

*Animals.* Six-months old, AKR male mice ($25^{+}2$ g) (provided by the Oncology Institute of Cluj-Napoca) were used in all experiments. The Ehrlich ascites tumor (Lettre variant) was maintained weekly by intraperitoneal injections of $2x10^6$ tumor cells per mouse.

*Compounds.* The two organoantimony(III) derivatives, *I.e.* (diphenylphosphinodithioato)di-phenylantimony(III), $Ph_2SbS_2PPh_2$ (compound **1**), and (di-isopropylphosphorodithioato)diphenyl-antimony(III), $Ph_2SbS_2P(OPr\text{-}i)_2$ (compound **2**), were prepared and purified as described earlier.[27,28] Both compounds were used at two concentrations (total dose of 10 and 20 mg/kg of body weight) in all the experiments.

*In vivo experiments.* Ten groups, each of ten animals (five groups of healthy mice and five groups of EAT-bearing mice) were used for the screening of the title compounds. The treatment was started 24 hours after the tumor transplant.

- *Experiment I*: Four groups of healthy animals were used for the administration of each organoantimony compound, at the two concentration levels. One group of healthy, untreated mice was considered as negative control (NC).

- *Experiment II*: Four groups of EAT-bearing mice were treated with the organoantimony derivatives, at the same concentration doses as above. One group of untreated, EAT-bearing mice was used as positive control (C).

The total doses of 10 or 20 mg /kg of the tested compounds were applied on days 1, 3 and 5 after tumor transplantation (day 0). For the healthy mice groups, the treatments were applied in the same days. All the animals were sacrified at 24 hours after the last treatment. Two hours before the sacrifice each mouse was injected with 1.5 ml colchicine 0.02%. Their bone marrow cells were collected from both femora by flushing with 2.5 ml MEM containing 20% fetal calf serum. The cell suspension from each experimental lot was partitioned in two aliquots, in order to apply the micronucleus test and the chromosomal analysis.

*Micronucleus test.* The cell suspension was centrifugated at 1500 rpm and the pellet was spread directly on slides, without fixation. The air-dried preparations were stained by the May-

Grünwald Giemsa method.[29] Two slides/mouse were prepared and examined microscopically. The polychromatic erythrocytes (PE) and micronuclei-containing PE (PEm), respectively, were counted from *ca.* 1000 erythrocytes. The results were expressed as the average number of PE (%) from total erythrocytes, and of micronucleated PEm (%) from one hundred PE. The statistical evaluation of the experimental *vs.* control data was calculated using the Student "t" test and the significance of the differences was set at 0.01 level.

*Chromosomal analysis.* The bone marrow cell suspension was centrifugated at 1500 rpm and the pellet was resuspended for 30 min. in a 0.7% natrium citrate hypotonic solution. The standard cytogenetic method was applied and finally the lymphoid cells were smeared on slides, air-dried and counted. For evaluation of the mitotic index (MI), 1000 cells from each slide were scored and the % of metaphases was recorded. Ten metaphases from each slide were examined, photographed and analysed in order to identify the number and the type of the chromosomal aberrations (chromatid or chromosome breaks and exchanges).

### RESULTS AND DISCUSSION

The mean percent values of polychromatic erythrocytes (%PE) from total erythrocytes and of micronucleated polychromatic (%PEm) from total PE, respectively, are presented in Table 1.

**Table 1.** Mean values (x), standard errors (+s) and statistical significance (S) of differences against controls (NC and C) for the parameters determined from the micronucleus test, after treatments with diphenylantimony(III) compounds (for the significance of the symbols, see *Materials and Methods*).

| Treatment | %PE | | | %PEm | | |
|---|---|---|---|---|---|---|
| (Exp-Cpd-Dose)[a] | x | ±s | S[b] | x | ±s | S[b] |
| NC | 42.5 | 1.5 | - | 0.30 | 0.03 | - |
| I - **1** - 10 | 40.0 | 1.1 | NS | 1.45 | 0.40 | * |
| I - **2** - 10 | 38.5 | 1.0 | NS | 2.50 | 0.72 | ** |
| I - **1** - 20 | 38.0 | 0.9 | * | 1.80 | 0.50 | ** |
| I - **2** - 20 | 36.5 | 0.8 | *** | 2.75 | 0.80 | ** |
| C | 48.6 | 2.8 | - | 0.55 | 0.05 | - |
| II - **1** - 10 | 48.0 | 1.8 | NS | 1.80 | 0.55 | NS |
| II - **2** - 10 | 45.0 | 1.3 | NS | 2.50 | 0.80 | NS |
| II - **1** - 20 | 40.0 | 1.1 | * | 2.80 | 0.95 | * |
| II - **2** - 20 | 36.0 | 0.8 | *** | 3.30 | 1.00 | * |

[a] Experiment (I or II) - Compound (**1** or **2**) - Dose (10 or 20 mg/kg); [b] * - $p < 0.01$, ** - $p < 0.005$, *** - $p < 0.001$.

A general decrease of %PE and an increase of %PEm compared to controls (NC and C) were observed for all the organoantimony-treated mice. These effects were dose-dependent and more pronounced for compound **2** than for compound **1**. Almost the same intensity of these effects was observed in healthy or Ehrlich tumor bearing mice.

Micronuclei originate from chromosomal material that has lagged in anaphase and was not included in the main nucleus of daughter cells. They are identified as dark-blue-staining bodies in the cytoplasm of PE which are coloured blue-pink, differently from mature normochromatic erythrocytes.[16] Figure 1 shows two different micronuclei identified from the Experiments I (**A**, I - **2** - 20) and II (**B**, II - **2** - 20), in which a dose of 20 mg/kg of compound **2** was used.

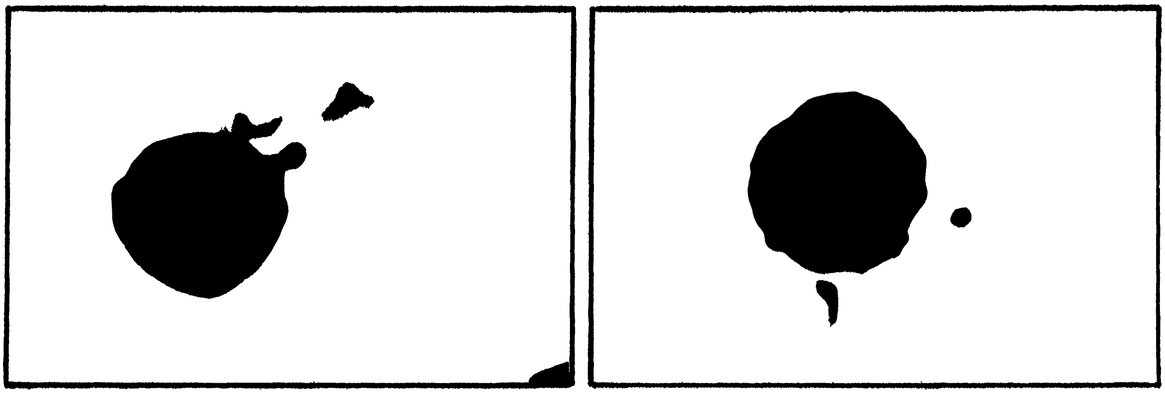

**Figure 1.** Micronuclei identified in polychromatic erytrocytes from healthy mice (**A**) and EAT-bearing mice (**B**) treated with 20 mg/kg of compound **2**.

The statistical evaluation of our results (the differences against controls - Table 1) revealed significant inhibition of PE at higher doses and significant stimulation of micronuclei formation in bone marrow cells from healthy mice, especially for compound **2**. In spite of these differences, it is difficult to design this compound as a strong mutagen. So far, the interpretation of the micronucleus test results remains difficult, the limit between a positive and a negative result being not yet established.[16] Generally, when the experimental values exceed ten times the control values, the tested compound is considered as a mutagen, but even this limit is not always accepted.[16,17]

Concomitently, the metaphase analysis was performed for the same experiments. Table 2 presents the mitotic index (MI) of bone marrow lymphocytes and the frequency of chromatid and chromosomal aberrations (breaks and exchanges). A general decrease in the mitotic index was observed in both experiments, the effect being dose-dependent and more pronounced when treatment with compound **2** was used. The most frequent aberrations of bone marrow cells were at chromatid level. The percentage of total aberrations was higher for compound **2**, especially in EAT-bearing mice. Figure 2 reveals two metaphases containing chromatidic breaks or exchanges, due to the administration of 20 mg/kg compound **2** to EAT-bearing mice.

**Table 2.** Mean values of the mitotic index (MI) and frequencies of chromosomal aberrations in bone marrow lymphocytes after the *in vivo* treatment with diphenylantimony(III) compounds (for the significance of the symbols, see *Materials and Methods*).

| Treatment (Exp-Cpd-Dose)[b] | MI | Chromatid aberrat.[a] breaks | Chromatid aberrat.[a] exchanges | Chromosome aberrat.[a] breaks | Chromosome aberrat.[a] exchanges | Total aberrat. (%) |
|---|---|---|---|---|---|---|
| NC | 0.08 | 2 | 0 | 0 | 0 | 2 |
| I - 1 - 10 | 0.05 | 1 | 0 | 1 | 0 | 2 |
| I - 2 - 10 | 0.04 | 3 | 1 | 0 | 0 | 4 |
| I - 1 - 20 | 0.05 | 2 | 1 | 1 | 0 | 4 |
| I - 2 - 20 | 0.02 | 4 | 2 | 2 | 0 | 8 |
| C | 0.15 | 5 | 1 | 1 | 0 | 7 |
| II - 1 - 10 | 0.12 | 4 | 2 | 1 | 0 | 7 |
| II - 2 - 10 | 0.10 | 5 | 2 | 2 | 1 | 10 |
| II - 1 - 20 | 0.10 | 5 | 1 | 2 | 1 | 9 |
| II - 1 - 20 | 0.09 | 8 | 3 | 2 | 1 | 14 |

[a]Mean values per 100 cells scored/slide; [b] The same abreviations as for Table 1.

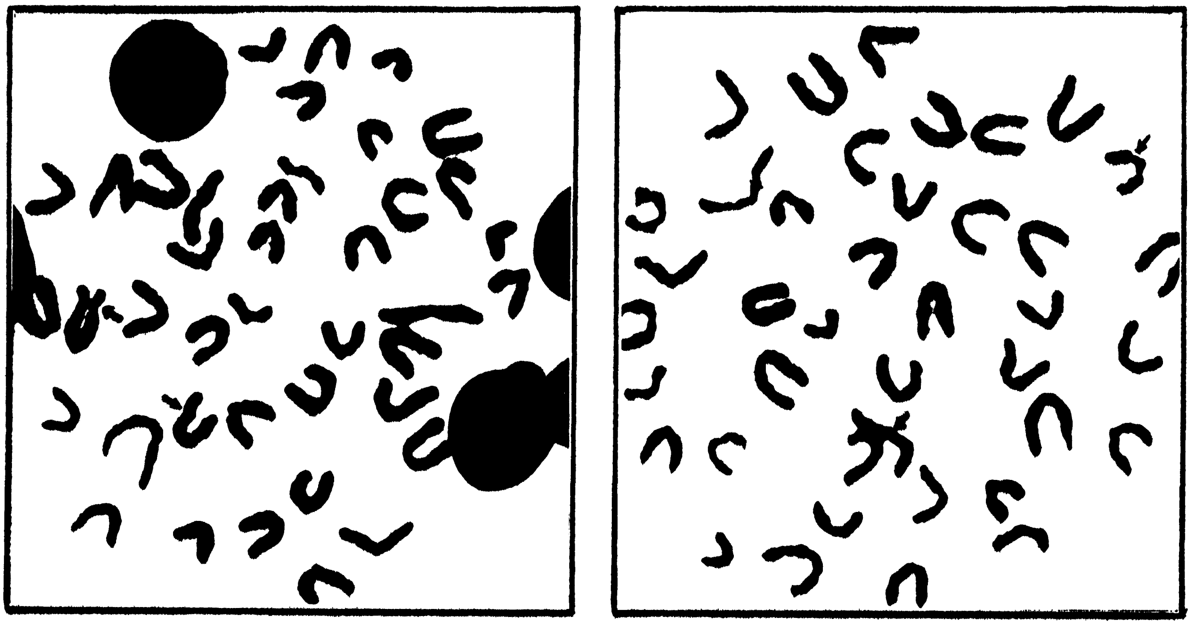

**Figure 2.** Metaphase chromosomes with chromatidic a berrations (breaks and exchanges *see the arrows*) obtained from lymphocytes of EAT-bearing mice, after treatment with 20 mg/kg of compound **2**.

Both methods, *i.e.* the micronucleus test and the metaphase analysis, are considered as equally sensitive for evaluating the mutagenicity. Most regulatory guidelines leave the choice to the investigator as to which of these tests to use for best-set screening.[16] We are considering that a simultaneous use of both methods might better reflect the real effect of the tested compounds.

Our results suggest that both compounds could have a mutagenic potential at the higher dose used (*i.e.* 20 mg/kg). This effect is more pronounced for (di-isopropylphosphorodithioato)diphenylantimony(III) (compound **2**), and is in good agreement with our previous observations concerning its cytotoxicity[23,24] and its genotoxicity evaluated using SOS Chromotest,[30] a well known bacterial test[15] for the identification of the mutagens.

### CONCLUSIONS

Our previous studies concerning the antitumor activity of the same two diphenylantimony(III) compounds revealed a stronger tumor growth inhibition produced by the phosphorodithioato derivative than the phosphinodithioato analogue. This activity was demonstrated *in vitro* and *in vivo* by an inhibition of tumor cell viability and proliferation, and an unbalance of ATP-producing and -consuming processes during the cell-cycle.

The present experiments have used as marker the bone marrow cells from healthy and EAT-bearing mice, and applied two short-term *in vivo* tests, *i.e.* the micronucleus test and the cytogenetic analysis, as end-points for mutagenicity. The results obtained from both tests compared well and revealed a mutagenic potential for both compounds used. The intensity of this effect was higher for the (di-isopropylphosphorodithioato)diphenylantimony(III). Another genotoxicity test, *i.e.* SOS Chromotest, confirmed the present results. It remains to be investigated how the results of these short-term tests could be correlated with long-term cancer studies. Further studies concerning this topic are in progress.

### REFERENCES

1. I. Haiduc and C. Silvestru, *Organometallics in Cancer Chemotherapy*, CRC Press, Boca Raton, Florida, Vol.I. Main Group Metal Compounds, 1989; Vol.II. Transition Metal Compounds, 1990.
2. M. Gielen (Ed.), *Metal-Based Anti-Tumour Drugs*, Freund Publ. House Ltd., London; Vol.1, 1988; Vol.2, 1992.
3. B.K. Keppler (Ed.), *Metal Complexes in Cancer Chemotherapy*, VCH, Weinheim (Germany), 1993.
4. A.M.J. Fichtinger-Schepman, A.T. Van Oosteron, P.H.M. Lohman and F. Berends, *Mutation Res.*, 1987, **190**, 159.

5. D. Wierda and T.L. Pazdernic, *Eur. J. Cancer.*, 1979, **15**, 1013.
6. L.A. Loeb and R.A. Zakour, in *Nucleic Acid-Metal Ion Interactions*, (T.G. Spiro, Ed.), J.Wiley & Sons, New York, 1978, p. 117.
7. T.C. Hsu, L.M. Cherry and S. Pathak, *Mutation Res.*, 1982, **93**, 185.
8. E. Bocian, M. Laverick and A.H.W. Nias, *Br. J. Cancer*, 1983, **47**, 503.
9. R.K.Y. Zee-Cheng and C.C. Cheng, *Meth. Find. Clin. Pharmacol.*, 1988, **10**, 67.
10. J.J. Roberts and M.F. Pera, Jr., in *Platinum, Gold and Other Metal Chemotherapeutic Agents: Chemistry and Biochemistry*, (S.J. Lippard, Ed.), ACS Symposium Series, 1983, **209**, 15.
11. F.W. Sunderman, Jr., in *Environmental Carcinogenesis*, (P. Emmelot and E. Kriek, Eds.), Elsevier, Amsterdam, 1979, p. 3.
12. A.V. Carrano and A.T. Natarajan, *Mutation Res.*, 1988, **204**, 379.
13. M. Ishidate, Jr., M.C. Harnois and T. Sofuni, *Mutation Res.*, 1988, **195**, 151.
14. J. Ashby, *Mutation Res.*, 1988, **204**, 543.
15. P. Quillardet and M. Hofnung, *Mutation Res.*, 1993, **297**, 235.
16. I.D. Adler, in *Mutagenicity Testing - A Practical Approach*, (S. Venitt and J.M. Parry, Eds.), IRL Press, Oxford, 1984, p. 275.
17. J.A. Heddle and A.V. Carrano, *Mutation Res.*, 1977, **44**, 63.
18. A.K. Sinha, V.A. Linscombe and B.B. Gollapudi, *Mutation Res.*, 1989, **226**, 65.
19. C. Socaciu, PhD Thesis, Babes-Bolyai University, Cluj-Napoca, Romania, 1986.
20. A.J. Crowe, *Drugs of the Future*, 1987, **12**, 255.
21. M. Gielen (Ed.), *Tin-Based Antitumor Drugs, NATO ASI Series: Cell Biology,* Springer Verlag, Berlin, 1990, Vol. 37.
22. C. Silvestru, C. Socaciu, A. Bara and I. Haiduc, *Anticancer Res.*, 1990, **10**, 803.
23. A. Bara, C. Socaciu, C. Silvestru and I. Haiduc, *Anticancer Res.*, 1991, **11**, 1991.
24. C. Socaciu, A. Bara, C. Silvestru and I. Haiduc, *InVivo*, 1991, **5**, 425.
25. B.K. Keppler, C. Silvestru and I. Haiduc, *Metal-Based Drugs*, 1994, **1** (in press).
26. G.R. Paton and A.C. Allison, *Mutation Res.*, 1972, **16**, 332.
27. C. Silvestru, L. Silaghi-Dumitrescu, I. Haiduc, M.J. Begley, M. Nunn and D.B. Sowerby, *J. Chem. Soc., Dalton Trans.*, 1986, 1031.
28. C. Silvestru, M. Curtui, I. Haiduc, M.J. Begley and D.B. Sowerby, *J. Organomet. Chem.*, 1992, **426**, 49.
29. N. Schmid, *Agents and Actions*, 1973, **3**, 77.
30. C. Socaciu, I. Pasca and O. Bobis, unpublished results.

**Received: October 27, 1993 - Accepted: January 10, 1994**

